# White-rot fungi scavenge reactive oxygen species, which drives pH-dependent exo-enzymatic mechanisms and promotes CO_2_ efflux

**DOI:** 10.3389/fmicb.2023.1148750

**Published:** 2023-06-08

**Authors:** Ignacio Jofré-Fernández, Francisco Matus-Baeza, Carolina Merino-Guzmán

**Affiliations:** ^1^Scientific and Technological Bioresource Nucleus (BIOREN), Universidad de La Frontera, Temuco, Chile; ^2^Laboratory of Geomicrobiology, Department of Chemical Sciences and Natural Resources, Universidad de La Frontera, Temuco, Chile; ^3^Laboratory of Conservation and Dynamics of Volcanic Soils, Department of Chemical Sciences and Natural Resources, Universidad de La Frontera, Temuco, Chile; ^4^Network for Extreme Environmental Research (NEXER), Universidad de La Frontera, Temuco, Chile

**Keywords:** white-rot fungi, SOM decomposition, greenhouse gasses, reactive oxygen species, enzymatic activity

## Abstract

Soil organic matter (SOM) decomposition mechanisms in rainforest ecosystems are governed by biotic and abiotic procedures which depend on available oxygen in the soil. White-rot fungi (WRF) play an important role in the primary decomposition of SOM via enzymatic mechanisms (biotic mechanism), which are linked to abiotic oxidative reactions (e.g., Fenton reaction), where both processes are dependent on reactive oxygen species (ROS) and soil pH variation, which has yet been studied. In humid temperate forest soils, we hypothesize that soil pH is a determining factor that regulates the production and consumption of ROS during biotic and abiotic SOM decomposition. Three soils from different parent materials and WRF inoculum were considered for this study: granitic (Nahuelbuta, *Schizophyllum commune*), metamorphic (Alerce Costero, *Stereum hirsutum*), and volcanic-allophanic (Puyehue, *Galerina patagonica*). CO_2_ fluxes, lignin peroxidase, manganese peroxidase, and dye-decolorizing peroxidase levels were all determined. Likewise, the production of superoxide anion (O_2_•-), hydrogen peroxide (H_2_O_2_), and hydroxyl radicals (•OH) were assessed in soils microcosms after 36 days of anaerobic incubation with WRF inoculum and induced Fenton reaction under pH variations ranging from 2.5 to 5.1. ROS significantly increased biotic and abiotic CO_2_ emissions in all tested soils, according to the findings. The highest values (217.45 mg C kg^−1^) were found during the anaerobic incubation of sterilized and inoculated soils with WRF at a natural pH of 4.5. At pH 4.0, the lowest levels of C mineralization (82 mg C kg^−1^) were found in Nahuelbuta soil. Enzyme activities showed different trends as pH changed. The Fenton reaction consumed more H_2_O_2_ between pH 3 and 4, but less between pH 4.5 and 2.5. The mechanisms that oxidized SOM are extremely sensitive to variations in soil pH and the stability of oxidant radical and non-radical compounds, according to our findings.

## Graphical Abstract

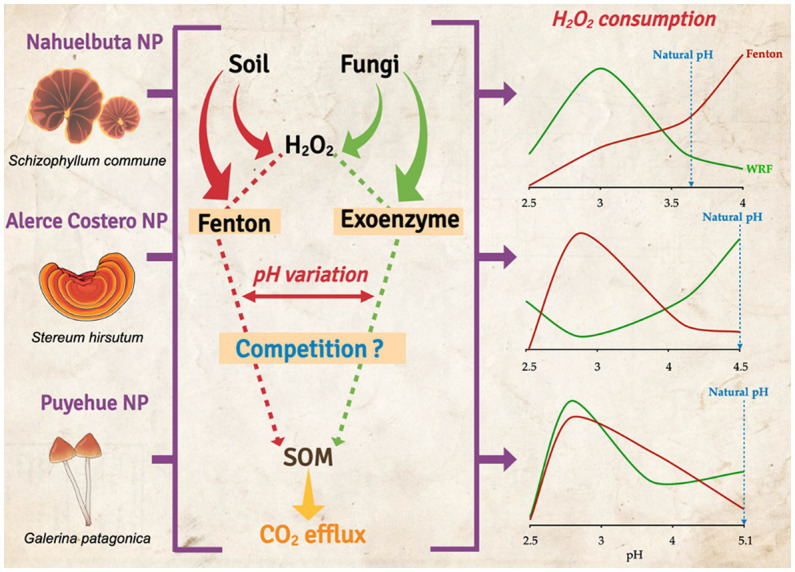

## 1. Introduction

The rate of industrialization has accelerated changes in carbon dioxide (CO_2_), methane (CH_4_), and reactive nitrogen oxides (NxO) emissions from human activities over the last 150 years (Köster et al., 2017; Walsh et al., 2017). Furthermore, the geological and natural sources from which they are released frequently contribute to the current climate change problem. After photosynthesis, soils are the second largest source of CO_2_ fluxes in terrestrial ecosystems (Raich and Schlesinger, 1992; Dixon et al., 1994; Schlesinger and Andrews, 2000). The sources of emissions are well understood, such as rhizosphere basal microbial respiration and the decomposition of plant residues and soil organic matter (SOM) (Kuzyakov, 2006). As a result, microbial communities play an important role in GHG emissions (Xu et al., 2013). Fungi have a significant function in the forest soil biome because they recycle and decompose both labile and recalcitrant materials like wood lignin under aerobic and anaerobic conditions (Lin et al., 2021; Naylor et al., 2022). This allows fungi to deal with changes in soil redox state resulting from rainfall in the winter and drying in the summer. This means that factors like soluble O_2_, pH, and oxidation–reduction processes in iron-rich soils in temperate rain forests may have a significant impact on SOM stability under a warming scenario.

The most common type of fungi in these rainforest soils that can efficiently convert lignin into CO_2_ are white-rot fungi (WRF). This fungus produces a diverse family of hemoperoxidases, including lignin peroxidases (LiP), manganese peroxidase (MnP), and dye peroxidase (DyP), all of which catalyze the decomposition of recalcitrant SOM via a step-by-step reaction catalyzed by hydrogen peroxide (H_2_O_2_) as acceptor, and resulting in CO_2_ efflux (Lenhart et al., 2012). The primary abiotic source of H_2_O_2_ in the soil is rainfall deposition (Willey et al., 1996; Kakeshpour et al., 2022). This occurs as a result of a photochemical or electrochemical reaction, such as water photolysis or an electrical storm (electro-Fenton). Furthermore, volatile terpene compounds can produce reactive oxygen species (ROS) in forest ecosystems via indirect biotic processes, reacting with ozone to produce hydroxyl radicals (•OH) and hydrogen peroxide (H_2_O_2_), both of which are then deposited in soil by precipitation (Becker et al., 1990). Furthermore, some aerobic soil bacteria produce biotic sources of ROS as a byproduct of respiration by releasing superoxide anion (O_2_•-), which is rapidly converted into H_2_O_2_ by the activity of different rhizosphere isoforms of extracellular superoxide dismutase enzymes (Takahashi et al., 2003; Jofré et al., 2021). Furthermore, ROS stability has been measured over a wide pH range (i.e., 2–10), whereas the maximum rate for metal oxidation occurs only at pH 3 (Garrido-Ramírez et al., 2010; Du et al., 2019).

Although SOM decomposition stimulates GHG release (Merino et al., 2020, 2021a), it is unclear whether biotic (exoenzymes) and abiotic (e.g., Fenton reaction) ROS production can be correlated with soil carbon emission, and whether this mechanism is transversal to the forest with similar structure on a not local scale. This hypothesis appears to be dependent on optimal pH values for biotic GHG production, which is dependent on enzymatic activity and abiotic pathways (Merino et al., 2021a). The optimal soil pH for methanogenesis, according to some reports, is between 4 and 7, but higher CO_2_ emissions were recorded at circumneutral pH (Dalal and Allen, 2008). As a result, soil pH affects all chemical, physical, and biological soil properties, affecting C fluxes.

Under pH variation, factors such as the availability and consumption of oxidant and radical species (H_2_O_2_, •OH, and O_2_•-) in biotic and abiotic redox reactions should be considered to determine the potential of GHG emissions in temperate rainforest soils. According to changes in soil pH, we hypothesize that the stability and consumption of reactive oxygen species (ROS) from biotic and abiotic reactions can significantly drive the CO_2_ emission rate from temperate rainforest soils. We investigated the dynamics of ROS under oxygen-limiting conditions, with and without white-rot fungal inoculum, and the Fenton reaction in long-term incubations in this study.

## 2. Materials and methods

### 2.1. Study sites and sampling

Three temperate forest soil types with mean annual precipitation ranging from 1,500 to >5,000 mm were chosen. The first soil sampled was a loamy Inceptisol (Soil Survey Staff, 2014) which was derived from intrusive granodiorite rocks in Nahuelbuta National Park (37°47′S, 72°12′W) (Bernhard et al., 2018). This soil was formed by ancient *Araucaria araucana* and Nothofagus pumilio forests. The second soil sampled in Alerce Costero National Park in the Coastal range under Nothofagus spp. and *Fitzroya cupressoides* (40°22′S 73°38′W) was a loamy clay Ultisol derived from metamorphic mica-schist materials with illite-kaolinite as dominant clays (Luzio et al., 2003). The final soil was collected in the Andes Cordillera from a primary temperate rainforest of Nothofagus betuloides in Puyehue National Park (40°47′S, 72°12′W) as Andisol derived from recent volcanic ash and basaltic scoria deposits with a high content of allophane, imogolite, and ferrihydrite (Neculman et al., 2013).

Four composite soil samples were extracted from the top Ah mineral horizon (0–15 cm) at each site after the litter layer was removed. The samples were cleaned in the laboratory to remove coarse organic debris and separated into two parts: one was stored at 4°C for a microcosm experiment and microbial analyses, and the other was air-dried for further physico-chemical analyses.

### 2.2. Analytical soil procedures

The pH and electrical conductivity were measured in an aliquot of soil in a 1:2.5 suspension of soil:water. TOC-VCSH (Shimadzu, Kyoto, Japan) was used to determine soil organic C, and total N was determined using Kjeldahl distillation (VELP, Usmate, Italy). The aluminum, iron and manganese were extracted by oxalate; Al_o_, Fe_o_ and Mn_o_ respectively, using 0.2 M ammonium oxalate at pH 3 (Sadzawka et al., 2006). The aluminium, iron and Mn complexed with SOM, extracted by pyrophosphate, Al_p_, Fe_p_ and Mn_p_, respectively were obtained using a solution of 0.1 M sodium pyrophosphate (Van Reeuwijk, 2002). To identify exchangeable, crystalline, and complexed-SOM metals in soil samples, dithionite-citrate-bicarbonate (Fe_d_) was used. Atomic absorption spectroscopy (Perkin Elmer 3110, Waltham, Massachusetts, United States) was used to determine Fe and Mn concentrations at 248.3 nm for Fe and 279.5 nm for Mn using a nitrous oxide acetylene flame. Cation exchange capacity (CEC) and nutrient characterization were carried out as Sadzawka et al. (2006) suggested. The total Fe concentration in 100 mg of dry soil was determined by adding 0.9 mL 0.28 M hydroxylamine hydrochloride and 1 mL 0.28 M HCl (Stookey, 1970). Approximately 100 μL of the extract was added to 4 mL of ferrozine color reagent (1 g ferrozine in 6.5 M ammonium acetate solution). Iron (II) concentration was determined in 100 mg of soil by adding 1 mL of 0.5 M HCl and vigorously shaking. Ferrozine reagent was added as previously described, and absorbance at 562 nm (ferrozine-Fe(II) complex standard) was measured in a UV spectrophotometer (Lovley and Phillips, 1987). The difference between total Fe and extractable Fe(II)-HCl forms was used to calculate the Fe(III) (oxyhydr) oxide content.

### 2.3. Culture conditions and fungal identification

In the same areas where soil samples were taken, white rot fungi were isolated from small fragments of decayed wood or fruiting body pieces. The fragments were placed in sterile tubes and stored at 4°C until they were analyzed. Small fragments of fungi fruiting bodies or decayed wood colonized by fungi were incubated on acidified glucose malt extract agar plates (15 g/L agar, 3.5 g/L malt extract, 10 g/L glucose, pH 5.5). Under aseptic conditions, pure mycelial cultures were obtained, and strains were identified using ITS sequencing. The DNA from each strain was extracted using the E.Z.N.A.® SP Fungal DNA Mini Kit D5524-01 (Omega, Bio-Tek, Norcross, GA, United States). The ITS1-5.8S—ITS2 rDNA was amplified using ITS1 and ITS4 primers (White et al., 1990). PCR was carried out in a total volume of 25 μL with 0.1 mM dNTPs, 0.1 mM of each primer, 5 U of Taq DNA polymerase, and the supplied reaction buffer (Promega Inc., Seoul, Korea). An ABI PRISM 3730 l DNA Analyzer System was used to sequence the PCR products (Macrogen, Seoul, Korea). The nucleotide sequences were compared in the GenBank database (Horisawa et al., 2013). Schizophyllum commune was identified in Nahuebuta soil, Stereum hirsutum in Alerce Costero soil, and Galerina patagonica in Puyehue soil (see Supplementary Table S1, for more information). In each microcosm, the isolates were inoculated at a final concentration of 3 × 10^8^ CFU gr soil^−1^ in 100 μL of sterilized water.

### 2.4. Microcosm anaerobic experiments

Under anaerobic conditions, the contribution of and ROS production and consumption from soils at different pH levels was determined in a sterilized portion of soil at field capacity (80%) from each soil (oxygen-free by N_2_ purge). In a destructive sampling design, soil samples were incubated in 120 mL serum bottles for 36 h at 12°C to monitor CO_2_ evolution, ROS production, and enzymatic activity. To remove the microbial resistant structures, the soil samples were sterilized for 20 min at 121°C (in an autoclave) on four consecutive days. The soils were then fumigated with chloroform vapor for 24 h in a vacuum chamber (Trevors, 1996). Autoclaving was chosen because it has little effect on the structure of SOM, primarily causing changes in the carbohydrate and N-alkyl domains (Berns et al., 2008). Because it causes Fe reduction and oxidation, gamma radiation was avoided (Bank et al., 2008; Abedini et al., 2014; Sutherland et al., 2017). Gamma radiation also increased the bioavailability of Fe(III) (oxyhydr)oxide minerals, which helped in Fe(III) reduction (Brown et al., 2012). There were three treatments (WRF, Fenton reaction, and WRF + Fenton) and four replicates with four induced pH in each soil; 2.5, 3, 4, and the natural pH of each soil (3.6, 4.5, and 5.1 for Nahuelbuta, Alerce Costero, and Puyehue, respectively). A H_2_O_2_:Fe(II) ratio was also added to induce the Fenton reaction, as previously reported by Merino et al. (2020). This was achieved by adding 120–143 mL of H_2_O_2_ (0.1 M) and 1.29 g Fe(II) kg^−1^ soil as FeCl_2_ to the soil (Sigma Aldrich, United States). A total of 144 serum bottles with septums for gas sampling were used. CO_2_ was collected at 0.5, 4, 8, 12, 24, and 36 days of incubation. Approximately 10 mL of CO_2_ gas sample was extracted and injected into a gas chromatograph with Flame Ionization Detector (GC/FID) (Thermo Fisher ScientificTM, Austin, TX) with a 30 mm DB1-MS column and selected ion mode at each sampling time. At the end of the incubation period, 72 microcosm bottles were harvested, and soil was homogenized and quickly subsampled for ROS analysis and enzymatic activity at each sampling.

### 2.5. Reactive oxygen species (ROS) detection

Superoxide anion (O_2_•-) production was measured in 0.5 g of soil, which was extracted with 12 mL of potassium phosphate buffer (PPB, 65 mM, pH = 7.8) and centrifuged at 10,000×*g* for 15 min at 4°C. The supernatant (5 mL) was combined with 0.9 mL of PPB, 1 mL of sulfanilamide (17 mM), 1 mL of hydroxylamine hydrochloride (10 mM), and 1 mL of naphthylamine (7 mM) and incubated in the dark for 20 min at 25°C. At 530 nm, the absorbance of the samples was measured (Greenwald, 2018). Hydrogen peroxide (H_2_O_2_) was determined using the iodometric titration method (Velikova et al., 2000). The concentration of H_2_O_2_ in mg L^−1^ was determined using a calibration curve as the standard. Although iodometric titration is less precise than permanganate titration, it produces fewer interferences with SOM (Liang and He, 2018; Merino et al., 2020). Page et al. (2013) previously used terephthalic acid (TPA) in soil to measure •OH production. Approximately 200 l of soil water (1:2.5 soil:water suspension) was injected into 3 mL aliquots of deoxygenated water for the blank (in triplicate) and 3 mL of TPA for the test (in triplicate) (1.5 mM, in triplicate). TPA-treated soil suspension was incubated for 24 h in the dark to oxidize into hydroxyterephthalic acid (hTPA). Finally, the sample was filtered at 0.22 m and the fluorescence was measured in a microplate reader at λex = 310 nm, λem = 425 nm (Synergy HT, Biotek). The fluorescence intensity was compared to a standard curve with 2-hydroxyterephthalic acid (0, 2, 20, 40, and 80 nM) (hTPA, Sigma Aldrich).

### 2.6. Enzyme activity

Exoenzymatic activities were spectrophotometrically measured under pH variations after 36 h of incubation. The formation of Mn(III)-tartrate complex during the oxidation of 0.1 mM MnSO_4_ in 100 mM tartrate buffer at pH 5 was used to determine manganese peroxidase (MnP) activity. At 238 nm, manganese peroxidase was measured spectrophotometrically (Xu et al., 2018). The oxidation of 2,2′-azino-bis (3-ethylthiazoline-6-sulfonate) (ABTS) (2.5 mM) to its cation radical in 100 mM tartrate buffer at pH 5 was followed by dye-decolorizing peroxidase (DyP) activity. At 418 nm, dye-decolorizing peroxidase was measured spectrophotometrically (Salvachúa et al., 2013). Veratryl alcohol (2 mM) was used as a substrate for LiP during the oxidation of 0.21 mL of 50 mM sodium tartrate buffer at pH 2.5. At 310 nm, lignin peroxidase was measured. The enzymatic activity tests were all performed in the presence of 0.1 mM H_2_O_2_. All oxidative enzymatic activities were measured in units (U) per milliliter (i.e., one millimole of substrate oxidized per minute). The measured H_2_O_2_ was normalized in relation to the treatment values in terms of representation.

### 2.7. Statistical analysis

The normal data distribution and variance homogeneity were tested for each treatment and soil type. One-way ANOVA was used for cumulative gas sampling (CO_2_). Using a repeated-measures ANOVA test, the enzyme activity, ROS production, and Fenton contribution were tested over a 40-day incubation period. The last three variables measured were plotted as a six-sample time average. Duncan’s multiple range test was used for multiple comparison means because all ANOVA tests were significant at p 0.05. The RStudio software was used for all analyses (1.1.442).

## 3. Results

### 3.1. Soil properties

The soils studied are formed from a variety of parent materials, including Granitic (Nahuelbuta), Metamorphic (Alerce Costero), and Volcanic-allophanic (Puyehue); their textures range from sandy to clay. The pH ranged from 3.6 to 5.1, with organic C levels ranging from 9.2 ± 0.1 to 11.4% ± 0.3. Natural H_2_O_2_ levels in soil range between 25.6 ± 0.7 and 33.7 ± 0.5 μM g^−1^ soil (Nahuelbuta<Alerce Costero< Puyehue soils). Puyehue soil had the highest content of Al complexed with SOM (Al_p_) (11 g kg^−1^ soil ±1.5), while Nahuelbuta soil had the lowest (0.7 g kg^−1^ soil ±0.1). The Al_o_ + 1/2 Fe_o_ > 2%, which indicates Andic properties <2, was found at higher levels of 3.8% in Puyehue soil. The Al_o_ symbol represents Al-oxides associated with amorphous structures, such as Alp with SOM. Puyehue soil had the highest value of 3.1 ± 0.2 g kg^−1^ soil. The Puyehue soil had the lowest CEC (5.3 cmol(+) kg^−1^), while Alerce Costero soil had the highest (19.7 cmol(+) kg^−1^). The total Fe content was determined by combining the soluble ferrous iron Fe(II) and ferric iron Fe(III) forms, which ranged between 8.4 ± 0.5 and 11.8 ± 0.5 g kg^−1^. Alerce Costero soil had a higher total Fe content than other soils (Table 1).

**Table 1:** Characteristics of soil used in the study.

Analysis	Units	Nahuelbuta	Alerce Costero	Puyehue
SOC^a^	%	9.20 ± 0.1	9.7 ± 0.2	11.4 ± 0.3
Total *N*	%	0.5 ± 0.01	0.4 ± 0.00	0.6 ± 0.03
C:N ratio	Unitless	24.3	23.8	19.1
pH water	Unitless	3.6 ± 0.2	4.5 ± 0.2	5.1 ± 0.1
H_2_O_2_	μM g^−1^ soil	25.6 ± 0.7	28.0 ± 0.9	33.7 ± 0.5
Al_p_^b^	g kg^−1^ soil	0.7 ± 0.1	5.7 ± 0.02	11.0 ± 1.5
Al_O_^c^	g kg^−1^ soil	7 ± 0.02	0.73 ± 0.1	3.1 ± 0.2
Al_o_ + 1/2 Fe_o_	Unitless	1.25	1.85	3.8
Al Saturation	%	80.0	93.5	22.4
Total Fe	%	10.4 ± 0.3	11.8 ± 0.5	8.4 ± 0.5
Fe^2+^	%	2.3 ± 0.5	5.8 ± 0.4	3.2 ± 0.2
Fe^3+^	%	8.1 ± 0.3	6.0 ± 0.5	5.2 ± 0.3
CEC^d^	cmol(+)kg^−1^ soil	11.8	19.7	5.3
Parent materials	Unitless	Granitic	Metamorphic	Volcanic-allophanic
Clay type^e^		K	Q,I,K	Allophane-imogollite
Texture^f^		L	CL	SCL

a Soil organic carbon.

b Pyrophosphate extractable Al.

c Oxalate extractable Al.

d Effective cation exchange capacity.

e Q quartz, K kaolinite, I illite.

f SCL sandy clay loam, CL clay loam, L loam.

### 3.2. CO_2_ evolution

**Figure 1:**
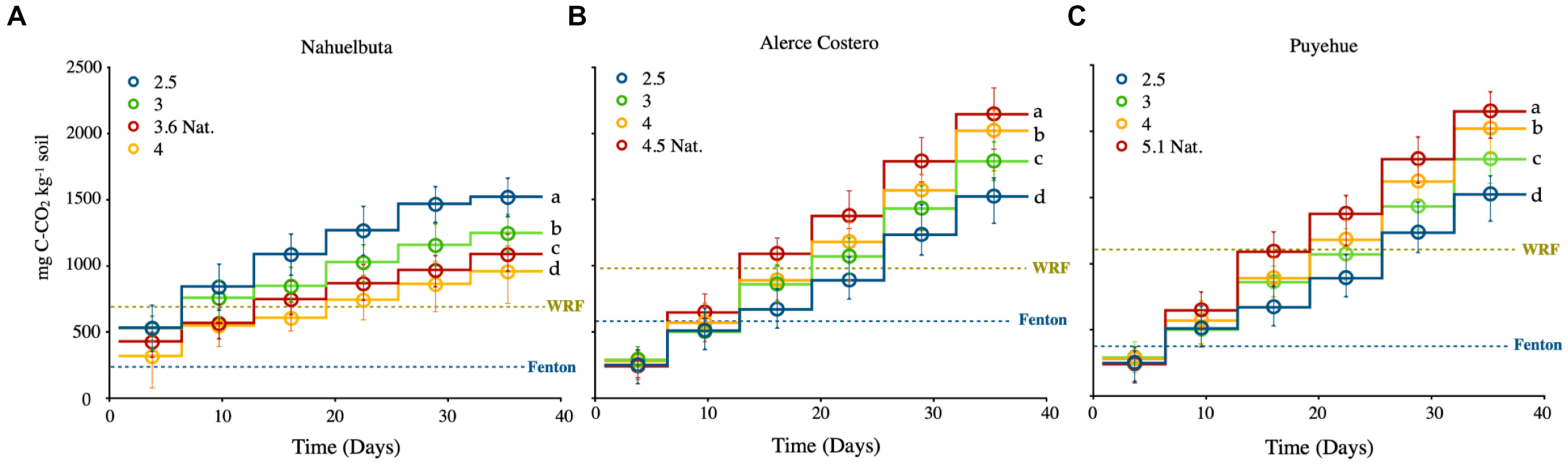
**(A–C)** CO_2_ evolution from anaerobic sterilized soils inoculated with WRF and H_2_O_2_/Fe for Fenton reaction, derived from three temperate rain forest sites with varying pH and incubated for 40 days at 12°C. The blue dotted lines represent the average rate of CO_2_ released by the induced Fenton reaction in sterilized soils at natural pH, while the green dotted lines represent the average rate of CO_2_ released by WRF at natural pH without the induction of the Fenton reaction. Significant differences (*p* < 0.05) are indicated by different letters in each panel. The ladder graph depicts the total amount of CO_2_ emitted between measurements.

Over the course of 36 days, increasing patterns of CO_2_ efflux were observed in all evaluated soils (Figures 1A–C). The highest values were found in Alerce Costero soil during anaerobic incubation of sterilized samples inoculated with WRF, at pH of 4.5 (217.45 mg C kg^−1^) (Figure 1B). The lowest levels of C mineralization (82 mg C kg^−1^) with the lowest pH 4 were found in Nahuelbuta soil (Figure 1A). Alerce Costero and Puyehue soils followed a similar pattern, with the highest CO_2_ release occurring at the highest pH levels, 4.5 and 5.1, and the lowest at induced pH 2.5 (Figures 1B,C). Figure 1A depicts the inverse trends for Nahuelbuta soil. Furthermore, all the soils treated with WRF without Fenton, and soils treated without inoculum, showed a lower average rate values than the combined treatments (WRF + Fenton). Similarly, the lowest average rate of mineralization was observed in sterile soils treated with Fenton, followed by WRF without Fenton induction (Figures 1A,C).

### 3.3. Exo-enzyme activities

**Figure 2:**
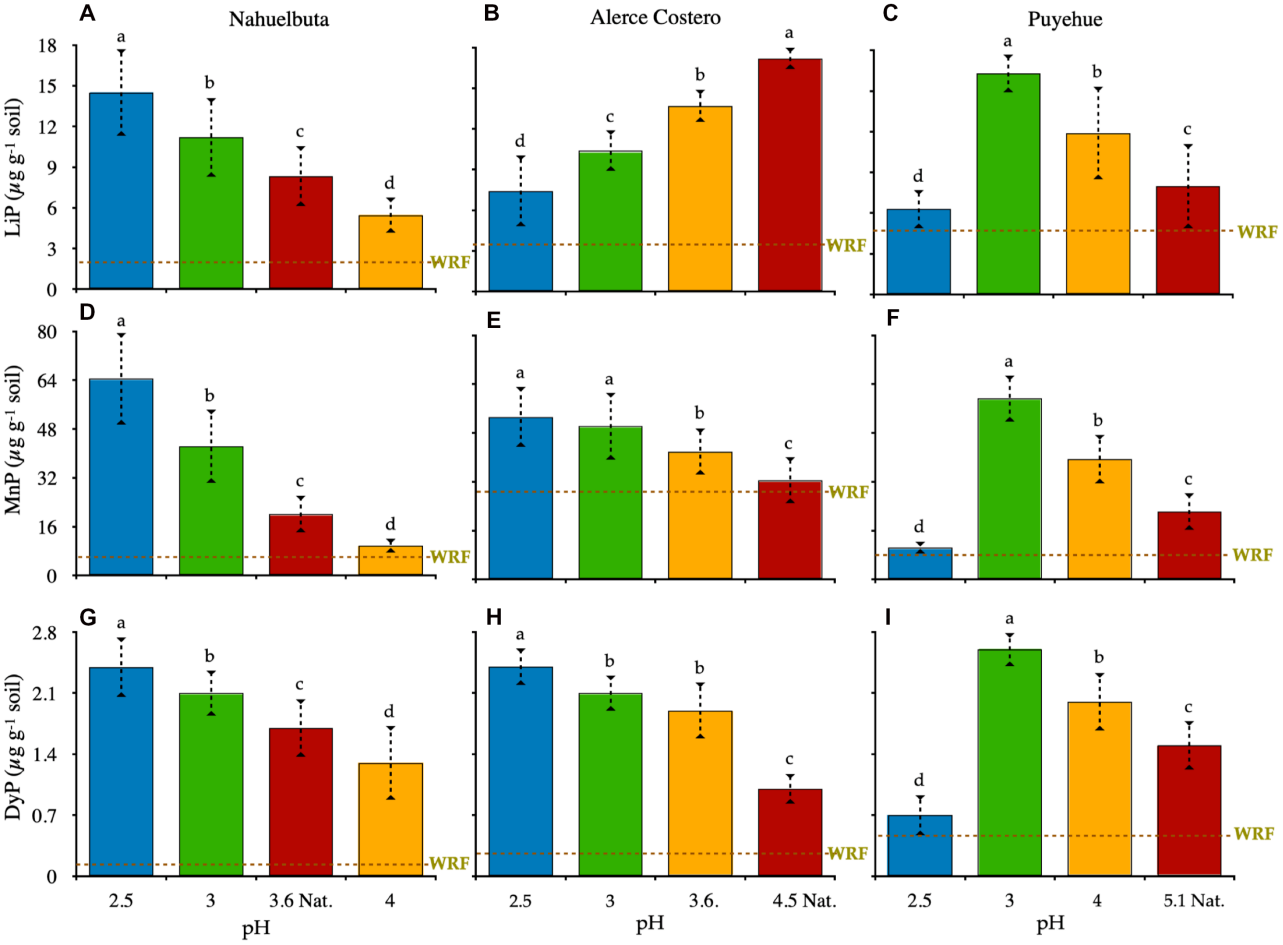
**A–C**: lignin peroxidase (LiP); **D–F**: manganese peroxidase (MnP); and **G–I**: dye-decolorizing peroxidase (DyP) in soils inoculated with WRF from three temperate forest soil sites at various pH levels. At natural pH, the green dotted lines represent the average rate of cumulative enzyme activity by WRF in the absence of the Fenton reaction. Within each panel, different letters indicate significant differences (*p* < 0.05).

After 36 days of incubation, the activity of LiP, MnP, and DyP was measured. When compared to non-induced Fenton groups, all WRF plus induced-Fenton reactions increased proportionally to enzyme activity. The responses of enzyme activities to pH changes in the soils studied varied (Figure 2). Except for Puyehue soil, only the Nahuelbuta microcosm showed a decrease in activity as the pH increased, with the highest activity observed at pH 2.5 (Figures 2A,D,G). LiP, MnP, and DyP were one, two, and eight times higher, respectively, than at natural pH. The CO_2_ released was distributed uniformly across the soil pH gradient. With the exception of Alerce Costero with LiP and Puyehue, the highest pH showed the lowest enzymatic activity and CO2 release after 36 days of incubation. LiP activity in Alerce Costero soil displayed a distinct pattern of activity when compared to MnP and DyP. The activity of the LiP increased as the pH increased from acidic to moderate acidic, with higher activity observed at pH (4.5) (Figure 2B). MnP and DyP, on the other hand, significantly reduced their activity from pH 2.5 to 4.5, while CO_2_ increased at day 36 (Figures 2E,H). The pH variation in Puyehue soil was observed to have a non-linear distribution. Lip, MnP, and DyP activity were found to be lower in acidic conditions (pH 2.5), with the highest activity at pH 3 (Figures 2C,F,I), followed by a decrease in activity at natural pH (4.5).

### 3.4. Effect of pH on *ROS* generation

**Figure 3:**
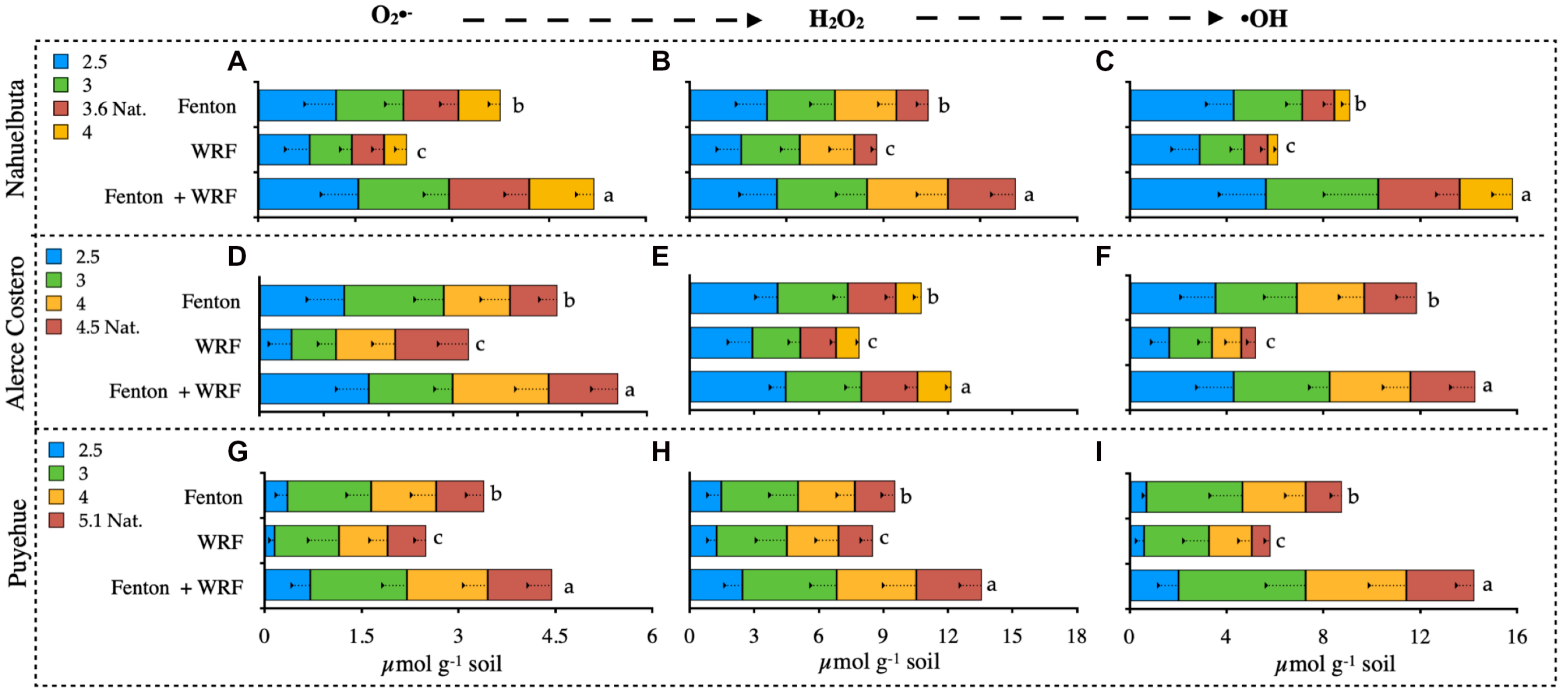
Detection of reactive oxygen species (ROS): superoxide anion **(A,D,G)**; hydrogen peroxide **(B,E,H)**; and hydroxyl radical **(C,F,I)** from soils inoculated with WRF from three temperate forest soil sites at different pHs. Significant differences (*p* < 0.05) are indicated by different letters in each panel.

pH had an effect on O_2_•- production, and the variation was significant between treatments and soils, ranging from 0.5 to 1.3 μmol g^−1^ soil (Figures 3A,D,G). The highest values in Nahuelbuta soil were obtained at higher pH, in the Fenton + WRF group, and in the independent treatments (Figures 3A–C). This pattern was also observed in Alerce Costero soil, where pH 2.5 and 3 had the highest levels of O_2_•-, decreasing to 4 and 4.5 (Figure 3A). In comparison to the other soils, the values in Puyehue soil were inverse to the trend (Figure 3G). The lowest levels of O_2_•- were found in the most acidic values (2.5), but at pH 3, the maximum production was reached, and as the pH increased, the O_2_•- content decreased (Figure 3G). As the pH changed, the production of H_2_O_2_ varied without a clear trend (Figures 3B,C,H). The highest value of 3.4 μmol g^−1^ of soil was discovered in Alerce Costero at a natural pH of 4.5 (Figure 3E). Puyehue had the lowest H_2_O_2_ production of 1.3 μmol g^−1^ soil and the lowest pH of 2.5 (Figure 3H). These values take into account the subtraction of the added H_2_O_2_ to initiate the Fenton H_2_O_2_/Fe(II) reaction (10,1 ratio). When compared to the other reactive oxygen species, the production of hydroxyl radicals (•OH) was the highest (ROS) (Figures 3C,F,I). Similar to H_2_O_2_ production, there was no discernible trend in •OH production, with the highest value of 4.3 μmol g^−1^ soil being recorded at the lowest pH 2.5 in Nahuelbuta (Figure 3C). The almost undetectable •OH 0.7 μmol g^−1^ soil was recorded in Puyehue with the lowest pH of 2.5 (Figure 3I). The WRF + Fenton treatment produced the highest total ROS, followed by Fenton and the WRF treatments with lower contributions (Figure 3). Furthermore, the cumulative production of ROS (the sum of the values of each ROS in all soils) was found to be lowest for O_2_•-, followed by •OH, and H_2_O_2_ had the highest abundance.

### 3.5. H_2_O_2_ consumption

**Figure 4:**
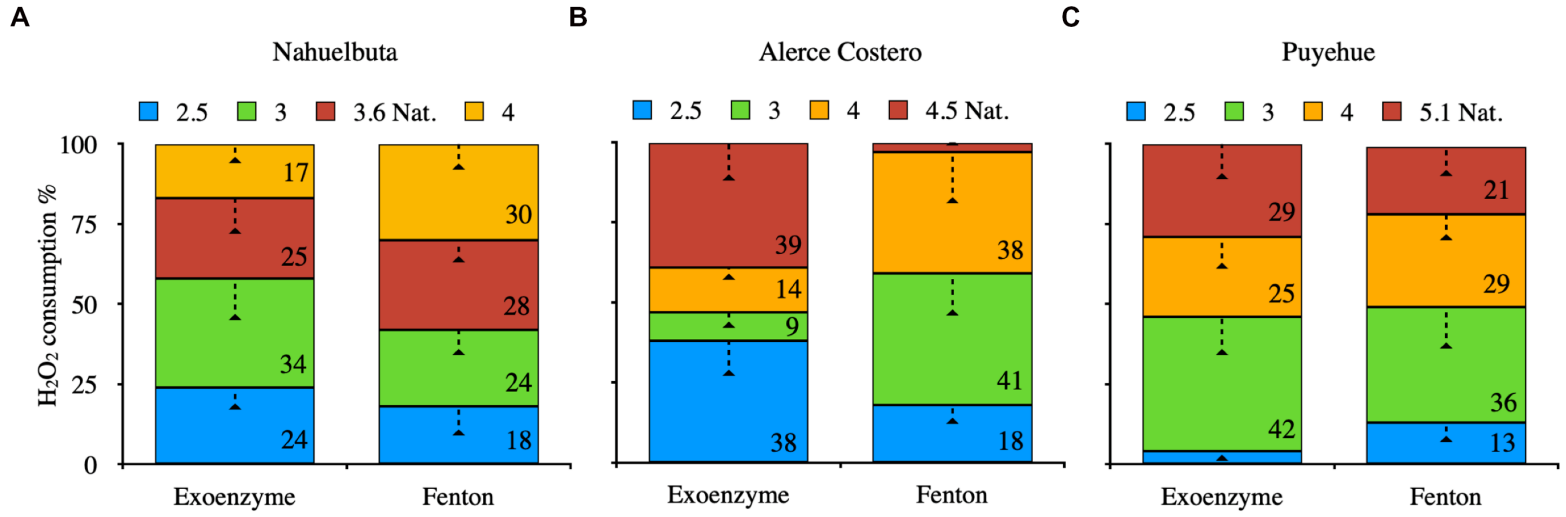
The average of exoenzyme activity or Fenton reaction from anaerobic sterilized soils inoculated with WRF from Nahuelbuta **(A)**, Alerce Costero **(B)**, and Puyehue **(C)** forest soil sites at different pH levels. Incubated for 36 days at 12°C. Significant differences (*p* < 0.05) are indicated by different letters in each panel.

Figure 4 depicts the consumption of H_2_O_2_ by exoenzyme and Fenton reaction after 36 days of incubation due to their high oxidative content, allowing them to be used as a cofactor in catalysis and iron oxidation (Fenton). When WRF was added to Nahuelbuta soil, this increased H_2_O_2_ consumption between pH 2.5 and 3.6, but decreased at pH 4. The Fenton reaction, on the other hand, consumes more H_2_O_2_ at higher pH levels (pH 4) (Figure 4A). This was not the case with Alerce Costero soil. At moderate acidity pH 4.5 and acid condition (2.5), the exoenzyme consumes more H_2_O_2_, whereas consumption is similar but significantly reduced at pH 3 and 4. The Fenton reaction, on the other hand, exhibits higher consumption values between pH 3 and 4, but lower consumption values between pH 4.5 and 2.5 (Figure 4B). At pH 3.6, similar consumption values were observed in both exoenzyme and Fenton reactions, but not at the other pH tested. When the pH is raised, exoenzymes consume the least. At pH 2.5, the Fenton reaction produced the lowest values (Figure 4C).

### 3.6. Relationships between variables measured

**Figure 5:**
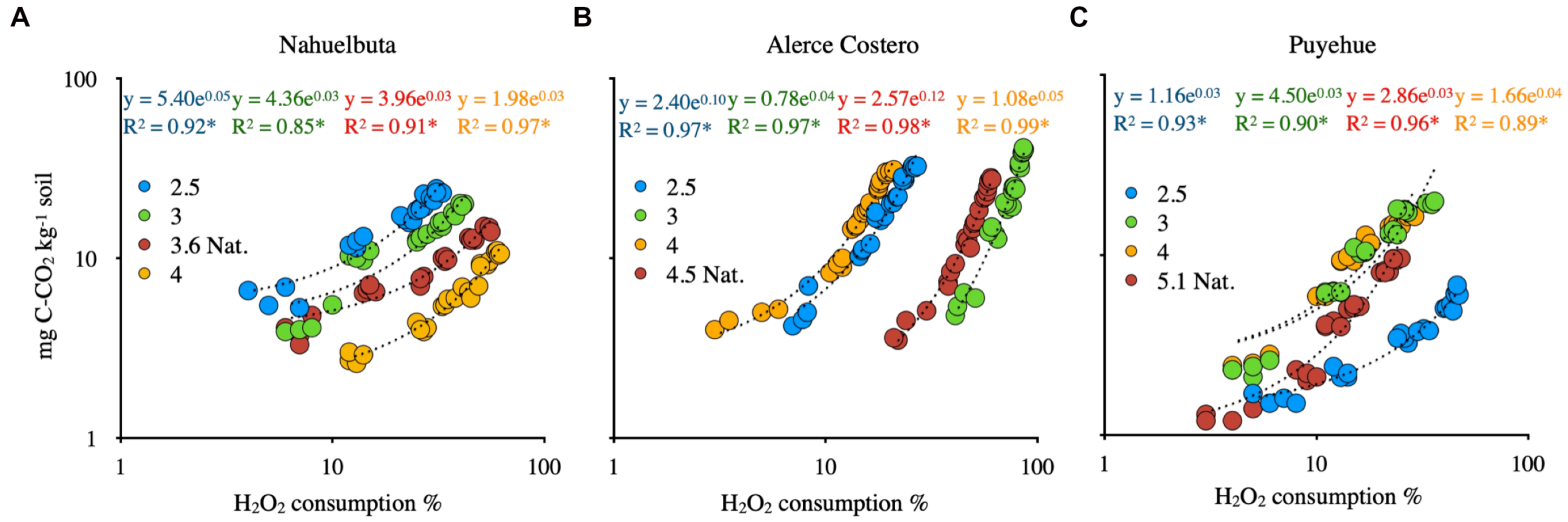
The relationships between CO_2_ and H_2_O_2_ consumption in anaerobic sterilized soils inoculated with WRF from Nahuelbuta **(A)**, Alerce Costero **(B)** and Puyehue **(C)** forest soil sites at different pH levels. For 40 days, it was incubated at 12°C. All relationships are statistically significant at *p* < 0.01.

The consumption of CO_2_ and H_2_O_2_ was found to have a positive and significant relationship (Figures 5A–C). CO_2_ production in WRF-treated soils was strongly related to peroxide consumption (*p* < 0.01, R2 > 0.85). Puyehue soil had a lower CO_2_ evolution trend associated with H_2_O_2_ consumption, but the tendency was lower in comparison to Alerce Costero soil, which had a higher CO_2_ production and H_2_O_2_ consumption at pH 3, with a steeper slope (Figures 5B,C).

## 4. Discussion

The abiotic decomposition of SOM is driven by reactive oxygen species such as O_2_•-, •OH, and H_2_O_2_, which occurs naturally even in the absence of enzymatic activity (Piccolo et al., 2018). H_2_O_2_ can be incorporated into soils via wet and dry atmospheric deposition (Petigara et al., 2002), intense solar radiation (Georgiou et al., 2015), oxidation of Fe (II) or reduced dissolved organic carbon (DOC) by oxygen (Page et al., 2013; Trusiak et al., 2018), and biotic input via the release of various oxidative compounds by soil bacteria and fungi. The biotic or abiotic H_2_O_2_ input is critical in the decomposition of labile and recalcitrant SOM, increasing H_2_O_2_ concentration (Merino et al., 2020, 2021b). An excess of H_2_O_2_, on the other hand, causes competition for H_2_O_2_ between abiotic and biotic processes.

The WRF generates endogenous H_2_O_2_ that serves as a cofactor for many enzymes that control fungal biomass conversion and the activity of lignolytic enzymes (e.g., Mn-peroxidase, LiP-peroxidase, lytic polysaccharide monooxygenases), which can be driven in the absence of O_2_ (Wong, 2009; Suryadi et al., 2022). Brown-rot fungi, which lack lignin peroxidases, may use the released H_2_O_2_ to drive the Fenton reaction, which is unique to these fungi (Arantes et al., 2012). As a result, competition can have a significant impact on the efficiency with which SOM is mineralized. Excess H_2_O_2_ may cause parallel (additive or synergistic) biotic and abiotic reactions, whereas fungi native production will compensate for the oxidant’s substrate for the Fenton reaction if there is insufficient H_2_O_2_. This would have an antagonistic effect on SOM degradation efficiency.

Both biotic (Diaz et al., 2013; Shah et al., 2016) and abiotic (Garrido-Ramírez et al., 2010; Georgiou et al., 2015) factors influenced the level of H_2_O_2_ in soil. The pH of the soil influences both the biotic and abiotic response to oxidative processes, according to our findings. The biotic component is most sensitive to changes in pH, whereas Fenton-mediated oxidation increases H_2_O_2_ consumption and CO_2_ efflux. When the pH was close to 5.1, the Alerce Costero and Puyehue soils showed similar higher CO_2_ emission trends. The highest efflux was observed in Nahuelbuta soil at more extreme acidic pH levels (2.5). This could be attributed to the *Schizophyllum commune* strain, which grows well *in vitro* under acidic conditions, which could translate into increased enzymatic activity. Microbial activity regulates extracellular O_2_•- concentrations in the soil environment (Diaz et al., 2016), which may affect soil H_2_O_2_ levels. The effect of pH treatment on WRF activity in the three soils studied was comparable to previous studies (Leff et al., 2015; Ling et al., 2016). Soil CO_2_ emissions had a significant positive relationship with soil H_2_O_2_ consumption (Figure 5). Increased biodiversity may improve Fe mobilization and H_2_O_2_ decomposition in soils, according to these findings. This effect is closely related to soil functional selection, which includes nutritional and physical conditions that favor the establishment of specific microorganisms over others (Mimmo et al., 2014). Based on soil organic matter (SOM) availability and N content, previous research has shown that endemic microbiota can cause significant CO_2_ flux and reactive oxygen species (ROS) production (Merino et al., 2021a).

Reactive oxygen species are known to play a critical role in the generation of CO_2_ by WRF (Hammel et al., 2002), the soil CO_2_ released was investigated using ROS as a factor. Given that H_2_O_2_ has long been used as an oxidant in soil degradation and SOM assessment, our discovery of a strong and linear relationship between CO_2_ released and soil H_2_O_2_ concentrations was not surprising (Robinson, 1927). Previously, researchers discovered a strong positive linear correlation between CO_2_ production and H_2_O_2_ concentrations in deciduous forest soils (Jugold et al., 2012). It is possible that different types of soil precursors will react differently to H_2_O_2_. More research on the specific precursors of abiotic CH_4_ produced in soils should be conducted to determine how the precursors and ROS are likely to interact biochemically. However, we were unable to establish a clear relationship between H_2_O_2_ concentrations and the other ROS precursors, implying that the H_2_O_2_ levels used in this study were possibly too high and that other factors (e.g., soil Fe or Mn) had become limiting in the generation of ROS.

Our findings show that anaerobic soils produce a higher proportion of •OH in soils, and that Fe(II) is the primary electron donor allowing the synthesis of •OH and subsequent SOM decomposition, which results in CO_2_ release in soils. •OH measurements during anaerobic incubation were consistent with previous research (Page et al., 2013; Minella et al., 2015). •OH generation in anaerobic and low-O_2_ soil can also be supported by reducing conditions (i.e., high electron donating capacity and thus high concentrations of electron donors) present across a broad pH range (Page et al., 2013). The pH and oxygen levels in the studied soils were significantly lower (Lipson et al., 2012, 2013), and Fe(II) concentrations provided highly reducing conditions. •OH production from the oxidation of reduced components such as Fe(II) or reduced SOM can be significantly influenced by pH. •OH can also be produced by oxidizing Fe(II) at a pH close to neutral (Remucal and Sedlak, 2011). For example, oxidation of Fe(II) at pH 5 may result in the formation of ferryl iron (Fe(IV)) and •OH, reducing the ratio of •OH generated per mol oxidized Fe(II) (Vermilyea and Voelker, 2009; Remucal and Sedlak, 2011). Other studies have revealed that at low pH (5), Fe(II) oxidation produces more •OH than Fe(IV) (Remucal and Sedlak, 2011). In contrast to Fe(II) oxidation, the effect of pH on the production of •OH from reduced DOC oxidation has not been studied. Aeschbacher et al. (2012) found that the oxidation of DOC to produce •OH is more favorable at high pH than at low pH, implying that this process occurs more frequently at higher pH.

## 5. Conclusion

This research investigated the impact of reactive oxygen species (ROS) on CO_2_ efflux and enzymatic activity during long-term anaerobic incubation. Despite the fact that few studies have proven that H_2_O_2_ availability has an effect, our findings show that abiotic and biotic reactions using reactive oxygen species as a feed for both mechanisms have an antagonistic and synergistic effect on GHG emission. However, in all soils tested, ROS production significantly increased biotic and non-biotic CO_2_ emissions. This implies that microorganisms and soil structure have an additional influence on C efflux, which is pH dependent. When bacterial microbiota undergoes redox fluctuations, WRF contributes more than 70% of GHG emissions (N_2_O and CO_2_), which is consistent with previous research conducted in the same forest region. According to the findings of this study, the mechanisms of microbial SOM oxidation are highly dependent on the stability and abundance of oxidant radical and non-radical compounds, as well as changes in soil pH.

## Data availability statement

The datasets presented in this study can be found in online repositories. The names of the repository/repositories and accession number(s) can be found below: NCBI—OQ341229, OQ339139 + OQ341421.

## Author contributions

CM-G and IJ-F: conceptualization, resources, writing—original draft preparation, and funding acquisition. CM-G: methodology, formal analysis, and project administration. IJ-F: investigation. FM-B: writing—review and editing. All authors contributed to the article and approved the submitted version.

## Funding

This research was funded by FONDECYT Postdoctorado, grant number 3200758, and FONDECYT regular, grant number 1220716. The APC was partially funded by Universidad de La Frontera, grant number DI21-1003.

## Conflict of interest

The authors declare that the research was conducted in the absence of any commercial or financial relationships that could be construed as a potential conflict of interest.

## Publisher’s note

All claims expressed in this article are solely those of the authors and do not necessarily represent those of their affiliated organizations, or those of the publisher, the editors and the reviewers. Any product that may be evaluated in this article, or claim that may be made by its manufacturer, is not guaranteed or endorsed by the publisher.

## Figures and Tables

**Table:** 

ROS	reactive oxygen species
WRF	White Rot Fungi
O_2_-	superoxide anion
H_2_O_2_	hydrogen peroxide
OH	hydroxyl radicals
SOM	soil organic matter
NxO	reactive nitrogen species
LiP	lignin peroxidase
MnP	manganese peroxidase
DyP	dye decolorizing peroxidase
GHG	green house gasses
Ah	first mineral horizon of soil
TOC	total organic carbon
CEC	cation exchange capacity
ITS	internal transcribed spacer
CFU	colony forming units
DOC	dissolved organic carbon
