# Supplementary material for: White-rot fungi scavenge reactive oxygen species, which drives pH-dependent exo-enzymatic mechanisms and promotes CO2 efflux
Source: Front Microbiol. 2023 Jun 8;14:1148750. doi: 10.3389/fmicb.2023.1148750 (PMC10285405; doi:10.3389/fmicb.2023.1148750)
Supplement: Supplementary file 1 [file Table_1.DOCX]

**Table S1.** Isolated white-rot fungi strains and ITS rDNA identification

| **Sample origin** | **Closest related species** | **Similarity (%)** | **Accession Number** |
| --- | --- | --- | --- |
| Nahuelbuta | *Schizophyllum commune* | 100 | AF249358.1 |
| Alerce Costero | *Stereum hirsutum* | 100 | AF506479.1 |
| Puyehue | *Galerina patagonica* | 99.61 | KM975403.1 |
